# Comprehensive insights on environmental adaptation strategies in Antarctic bacteria and biotechnological applications of cold adapted molecules

**DOI:** 10.3389/fmicb.2023.1197797

**Published:** 2023-06-16

**Authors:** Kesava Priyan Ramasamy, Lovely Mahawar, Raju Rajasabapathy, Kottilil Rajeshwari, Cristina Miceli, Sandra Pucciarelli

**Affiliations:** ^1^Department of Ecology and Environmental Science, Umeå University, Umeå, Sweden; ^2^Department of Plant Physiology, Faculty of Agrobiology and Food Resources, Slovak University of Agriculture, Nitra, Slovakia; ^3^Department of Marine Science, Bharathidasan University, Tiruchirappalli, Tamilnadu, India; ^4^Department of Zoology, Goa University, Taleigão, Goa, India; ^5^School of Biosciences and Veterinary Medicine, University of Camerino, Camerino, Italy

**Keywords:** Antarctic bacteria, climate change, psychrophiles, omics, machine learning, biotechnological applications

## Abstract

Climate change and the induced environmental disturbances is one of the major threats that have a strong impact on bacterial communities in the Antarctic environment. To cope with the persistent extreme environment and inhospitable conditions, psychrophilic bacteria are thriving and displaying striking adaptive characteristics towards severe external factors including freezing temperature, sea ice, high radiation and salinity which indicates their potential in regulating climate change’s environmental impacts. The review illustrates the different adaptation strategies of Antarctic microbes to changing climate factors at the structural, physiological and molecular level. Moreover, we discuss the recent developments in “omics” approaches to reveal polar “blackbox” of psychrophiles in order to gain a comprehensive picture of bacterial communities. The psychrophilic bacteria synthesize distinctive cold-adapted enzymes and molecules that have many more industrial applications than mesophilic ones in biotechnological industries. Hence, the review also emphasizes on the biotechnological potential of psychrophilic enzymes in different sectors and suggests the machine learning approach to study cold–adapted bacteria and engineering the industrially important enzymes for sustainable bioeconomy.

## Introduction

Approximately 70% of the Earth’s surface is covered by ice which includes ice caps, ice sheets, glaciers, sea ice and high mountain ranges (Yusof et al., 2021). Antarctica, the least populated continent placed at the southernmost of the Earth, contains about 90% of the world’s ice and is continually blanketed in ice sheets. Around 15% part of the continent is covered by sea ice. However, the ongoing climate change and stratospheric ozone consistently impact the continent and the residing organisms by rapidly altering environmental factors including temperature, precipitation, UV radiation etc. (Yusof et al., 2021). Climate change is influenced by anthropogenic emissions of greenhouse gasses (GHGs), which result in an increase in global temperature of up to 1.5°C or even 2°C (IPCC, 2021). The increase in average temperature leads to the amplification of permafrost thawing, melting of the Antarctic ice sheet (Dietz and Koninx, 2022), and changes in ice mass (Stokes et al., 2022). An increase in Antarctic ice sheet loss enhances the penetration of light and energy levels into water that changes plankton productivity and composition, including microbes (Barnes et al., 2021). Hence, climate-induced disturbances are major threats to the Antarctic ecosystem, especially to microbial composition and functions (Gutt et al., 2021).

Microbes are important components that generate the base of polar food webs in both terrestrial and freshwater Antarctic ecosystems, hence affecting all trophic levels. They play a crucial role in the biogeochemical cycle (especially in carbon and nitrogen cycling) and functioning of an extreme Antarctic environment (Anesio et al., 2009). Microbes thriving in polar cryosphere, which comprises 14% of the Earth’s surface for more than 33 million kilometers (Malard and Pearce, 2018) are called psychrophilic *sensu stricto* and psychrotolerant organisms, based on the optimum growth temperature. The minimum, optimum and maximum growth temperatures for psychrophiles are <0, <15 and < 20°C, and for psychrotrophs >0, >20 and > 30°C, respectively (Moyer and Morita, 2007). To persist in extreme environment and inhospitable conditions, these microbes are thriving and displaying striking adaptive characteristics towards severe external factors including low humidity, precipitation, freezing temperature, sea ice, high radiation and salinity, nutrients limitation and strong winds (Feller and Gerday, 2003). This indicates the potential of polar microbes in regulating climate change’s environmental impacts and represents their distinctive adaptability of primitive life-forms. Therefore, it is crucial to find how Antarctic microorganisms adapt to different climate-induced environmental stresses.

Several previous studies reported that distinctive adaptive characteristics of psychrophiles are due to the specific protein adaptations and enzymes they secrete (Kohli et al., 2020). The different types of bonding (covalent and H-bond), amino acid composition, G + C content and folding pattern of proteins are responsible for the improved stability and adaptation of psychrophiles (Dutta and Chaudhuri, 2010). However, due to the difficulty in direct cultivation of psychrophiles from extreme Antarctic environment, the research on their adaptation is still on the elementary phase and most of the adaptation strategies and their underlying mechanisms (freezing tolerance/avoidance, regulation of protein synthesis and membrane fluidity) have not been fully understood.

In this review, we focus on the different adaptation strategies of Antarctic marine bacteria to environmental changes at morphological, physiological, and molecular level. Moreover, the review highlights the biotechnological applications of Antarctic bacteria and their enzymes and sourced proteins in different industries. To unlock “blackbox” of Antarctic bacteria and engineer cold adapted molecules including psychrozymes, in the last section of the review, we discussed the possible use of recent technologies such as omics and machine learning as an effective, fast and less labor- intensive approach. Additionally, we emphasize the existing research gaps and illustrate our view on future research directions on Antarctic microbes, an unexplored ecosystem.

## Adaptation strategies of Antarctic bacteria

The adaptation of psychrophilic bacteria to the multitude of environmental stressors including low temperature is achieved via complex range of structural and physiological changes (Table 1) during long term evolution (Collins and Margesin, 2019). Several new emerging omics techniques including genomics, transcriptomics, metagenomics, metabolomics, etc. have increased our understanding of bacterial cold adaptation. These technologies demonstrated that psychrophilic bacteria employ various survival strategies to cope with the challenges confronted in their habitat. The following section includes the strategic tools adapted by bacteria at morphological, physiological and molecular level to enable life in the harsh Antarctic environment.

**Table 1:** Adaptation strategies in Antarctic bacteria in response to different stress environment.

Environmental conditions	Bacterial adaptation	Taxa	Reference
Temperature	Membrane fatty acids	*Flavobacterium* spp.	Králová (2017)
	EPS production	*Pedobacter polysacchareus* sp.	Wang et al. (2023)
	Transcriptional changes	*Pseudoalteromonas haloplanktis* TAC125	Riccardi et al. (2023)
	Expression of diguanylate cyclase (DGC) gene, biofilm formation	*Rhodococcus* sp. NJ-530	Wang et al. (2022)
	Chemical modifications of the lipopolysaccharide (LPS)	*Pseudoalteromonas tetraodonis* strain SY174, *Psychromonas arctica* strain SY204b, *Psychrobacter cryohalolentis* strain SY185	Di Lorenzo et al. (2020)
	Increase of saturated fatty acid	*Rhodococcus* sp. JG-3	García-Descalzo et al. (2023)
	Hydrolases, a clp protease, and novel YraN family endonuclease upregulation	*Cryobacterium* sp. SO1	Teoh et al. (2021)
	GH42 β-galactosidase	*Marinomonas* ef1	Mangiagalli et al. (2021)
	Ice-binding proteins (IBPs)	*Flavobacterium frigoris* PS1, *Nostoc* sp. HG1	Kim et al. (2019), Raymond et al. (2007)
	Extracellular polymeric substrates	*Nostoc* sp. strain SO-36	Effendi et al. (2022)
	Endolysins	*Pseudomonas* ef1	Orlando et al. (2020)
	Changes in the gene expression profile	*Shewanella baltica*	Kloska et al. (2020)
	Polyhydroxyalkanoates synthesis	*Pseudomonas* sp. MPC6	Orellana-Saez et al. (2019)
Salinity	Variation in fatty acids types composition	*Rhodococcus* sp. JG-3	García-Descalzo et al. (2023)
	Intracellular laccase-like protein	*Halomonas* sp. strain M68	Bisaccia et al. (2023)
	Biofilm formation, expression of diguanylate cyclase (DGC) gene	*Rhodococcus* sp. NJ-530	Wang et al. (2022)
UV	Pyomelanin production	*Pseudomonas* sp. ANT_H4	Styczynski et al. (2022)
	Role of DEAD-box RNA helicase	*Pseudomonas syringae* Lz4W	Hussain and Ray (2022)
	Production of polyhydroxyalkanoates	*Pseudomonas extremaustralis*	Tribelli et al. (2020)
	DNA photolyase	*Rhodococcus* sp. NJ-530	He et al. (2021)
	Production of carotenoids	*Planococcus* sp. ANT_H30, *Rhodococcus sp*. ANT_H53B	Styczynski et al. (2020)
	Expression of light reactive proteins	*Hymenobacter nivis* P3T	Terashima et al. (2019)
	Pigment production	*Hymenobacter* sp. *strain* UV11	Marizcurrena et al. (2019)
Hydrocarbon	Bio emulsifier production	*Paenibacillus antarcticus* IPAC21	de Lemos et al. (2023)
	Trinitrotoluene metabolic genes, xenobiotic reductases	*Pseudomonas* sp. TNT3, TNT11, and TNT19	Cabrera et al. (2020), Cabrera et al. (2022)
	Biosurfactant	*Bacillus* sp. ANT_WA51	Krucoń et al. (2023)
	Contains genes for aromatic compounds degradation	*Pseudomonas* sp. MPC6	Orellana-Saez et al. (2019)
Phenol	Phenol degradation	*Rhodococcus* sp. strain AQ5-14	Tengku-Mazuki et al. (2020)
Polyethylene	Laccase catalytic structure	*Psychrobacter* sp. NJ228	Zhang et al. (2022)
Heavy metal pollution	Increasing antioxidant enzymes (Super oxide dismutase, glutathione reductase) and antioxidant substances (glutathione, carotenoid)	*Planococcus* sp. O5	Cheng et al. (2022)
	Molybdenum reduction	*Arthrobacter* sp. *Strain* AQ5-05	Darham et al. (2021)
	Heavy metal tolerance genes	*Dietzia psychralcaliphila* JI1D	Ausuri et al. (2022)
Nutrients	Carbon metabolism	*Arthrobacter*	Gushgari-Doyle et al. (2022)
	Biofilm formation	*Pseudoalteromonas haloplanktis* TAC125	Ricciardelli et al. (2019)
	Production of extracellular hydrolytic enzymes	*Hymenobacter* sp. *strain* UV11	Marizcurrena et al. (2019)
High hydrostatic pressure	Antioxidant defenses and energy regulation	*Halomonas titanicae* ANRCS81	Li et al. (2023)
	Metabolic adaptation	*Microbacterium sediminis*	Xiao et al. (2023)

## Structural adaptation

**Figure 1:**
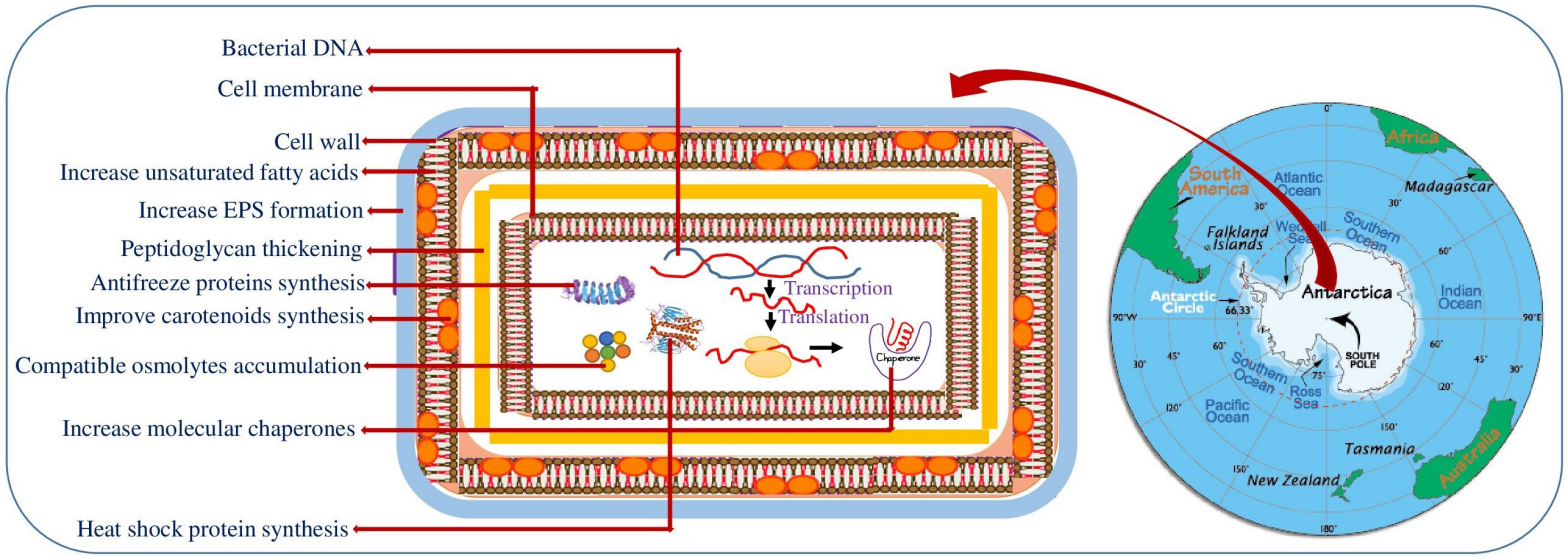
Schematic representation of the various cold adaptation strategies of Antarctic bacteria. Map of Antarctica retrieved from worldatlas.com.

The cell envelope is a complex multi-layered (outer, peptidoglycan and inner membrane) structure of bacteria that provides protection from the unpredictable and hostile environment. Several studies reported the thickening of outer cell surfaces in particular, peptidoglycan layer (Figure 1) (gram positive) and lipopolysaccharide (gram negative bacteria) in cold adapted bacteria (Di Lorenzo et al., 2020). The thick cell surface strengthens the psychrophiles against cell disruption by ice formation, increased osmotic pressure and freezing/thawing at low/subzero temperature (Figure 1). Recent study on the structural elucidation of the highly heterogenous lipid A (LPS’ glycolipid moiety) in the three Antarctic bacteria strains *Pseudoalteromonas tetraodonis* SY174, *Psychromonas arctica* SY204b, and *Psychrobacter cryohalolentis* SY185 showed that structural alterations in the LPS’ glycolipid moiety increase the membrane flexibility and stability of these psychrophiles in Antarctica (Di Lorenzo et al., 2020). To tolerate the negative effect of temperature stress, bacteria alter the composition of cell membrane fatty acids (Hassan et al., 2020). Cold-adapted bacteria usually increases the polyunsaturated to saturated fatty acid ratio in membrane phospholipids to maintain optimal fluidity and membrane permeability (homeoviscous adaptation) (De Maayer et al., 2014). In this regard Králová (2017) studied the role of membrane fatty acids in adaptation of *Flavobacterium* sp. towards harsh Antarctic environment (Table 1). The study suggested that Antarctic *Flavobacterium* sp. mostly utilizes two mechanisms of homeoviscous adaptation in cold-adaptive response- unsaturation of fatty acids and synthesis of branched fatty acids (Králová, 2017). Similarly, Bajerski et al. (2017) investigated the biomembrane polar fatty acid adaptation of *Chryseobacterium frigidisoli* PB4T isolated from an Antarctic glacier in response to varying temperature (0°C to 20°C) and pH (5.5 to 8.5). Another strategy adopted by the Antarctic bacteria is the production of extracellular polymeric substances (EPSs) that protects the cell from sub-freezing temperature by forming a protective covering around the bacterial cell that act as a barrier to solutes diffusion and ice formation (Collins and Margesin, 2019) (Figure 1). In this context, Caruso et al. (2018) studied the EPS synthesis in four Antarctic sponge-associated bacteria (*Shewanella* sp. strain CAL606, *Colwellia* sp. strain GW185, and *Winogradskyella* sp. strains CAL384 and CAL396). EPS production by psychrophilic bacteria has an ecological role in cell adhesion to surfaces and cell protection due to their chemical composition (Lo Giudice et al., 2020). In addition, EPS also plays a vital role in biofilm formation that enhances access to nutrients and cell survivability. The adaptation of haloarchaea *Halorubrum lacusprofundi* in Deep Lake (Antarctica) is a good example of cold adaptation by biofilm (Liao et al., 2016). Moreover, EPS secreted from cold adapted microbes shows ice binding functions and ice recrystallization inhibitors (IRI) activity (Casillo et al., 2017). Additionally, cold adapted microbes produce a substantial amount of biosurfactants that play a potential role in their survival.

## Physiological adaptation

Psychrophilic bacteria developed several physiological adaptations such as metabolic alterations, synthesis of pigments, compatible osmolytes and ice binding proteins to optimize their metabolism in harsh Antarctic environment (Figure 1) (Collins and Margesin, 2019). Pigment formation is a common feature in cold-adapted microbes thriving in diverse habitat including glaciers, high altitude, marine water and ice cores (Pandey et al., 2018). Pigments, in particular polar carotenoids, have been suggested to serve in maintaining the membrane fluidity and rigidity. Moreover, these pigments act as a photoprotector, antioxidants, cryoprotectants, antimicrobials and light harvesters in psychrophilic microbes to counteract the low temperature and other environmental stresses (Pandey et al., 2018). The pigment production by cold adapted bacteria including Antarctic environment have been extensively reviewed by Sajjad et al. (2020). Accumulation of compatible osmolytes (glycine betaine, trehalose, glycerol, sucrose, mannitol etc.) is the other way of Antarctic bacteria to prevent cell shrinkage and water loss during sub-zero temperature (Goordial et al., 2016). Improved concentration of compatible osmolytes restore osmotic balance by reducing the freezing point of the cell cytoplasm (Fonseca et al., 2016). The osmolytes were also found to be involved in scavenging free radicals, counteracting protein aggregation, improving protein folding and stabilizing membranes and proteins at chilling temperatures (Collins and Margesin, 2019).

Additionally, psychrophiles synthesize several proteins namely ice binding, cold shock and heat shock proteins to sustain their physiological state in changing environment (Figure 1) (Kim et al., 2018). Ice binding proteins are antifreeze proteins (AFPs) that on binding to ice inhibits the growth of ice crystals and lower freezing temperature. The proteins were initially reported in Antarctic fish and in various cold organisms including bacteria. Bar Dolev et al. (2016) identified the multidomain ice adhesion AFP in the Antarctic bacterium *Marinomonas primoryensis*. AFPs causes thermal hysteresis (TH) in which the freezing temperature of water drops below the melting temperature. This ceases ice growth by creating a thermal hysteresis gap (Mangiagalli et al., 2017). Antifreeze proteins are also found to display ice recrystallisation inhibitors (IRI) activity. A study by Raymond et al. (2007) suggested the role of AFPs isolated from Antarctic Gram-negative bacteria *Colwellia*, strain SLW05 in ice recrystallization inhibition and ice-binding. The cold shock proteins are a family of widely distributed low molecular weight highly conserved proteins, synthesized under normal environment but strongly induced when exposed to cold conditions. The cold shock gene homologous to *cspA* of *E. coli* has been isolated in two isolates of Antarctic psychrotrophs- Gram-positive bacterium *Arthrobacter protophormiae* and Gram-negative *Pseudomonas fluorescens* which expressed constitutively at two different temperatures (4°C and 22°C) (Ray et al., 1994). Kim et al. (2007) characterized cold shock protein A of the Antarctic bacterium *Streptomyces* sp. AA8321 and depicted its role in inhibiting DNA replication during cold adaptation. Similarly, characterization and expression of three cold shock protein (CSP) genes under different stress conditions is studied in the Antarctic bacteria *Psychrobacter* sp. G (Song et al., 2012). A comparative proteomic study of cold-repressed proteins in the *Pseudoalteromonas haloplanktis* TAC125 located in Antarctic environment at 4°C and 18°C demonstrated that majority of these proteins expressed at 4°C were heat shock proteins associated to folding (Piette et al., 2011). Furthermore, the increase accretion of heat shock proteins has been observed in Antarctic psychrophiles during ocean warming and acidification. Recently, Yusof et al. (2021) characterized two Hsp70 genes in the Antarctic yeast, *Glaciozyma antarctica* PI12 that protect the functional activity of the yeast under different temperature stress (4°,15°, 25 ° and 37°C). The Antarctic ciliated protozoon *E. focardii* maintains a constitutive synthesis of some Hsp70 genes to preserve protein functions in the cold and induces high expression of other Hsp70 genes in response to the oxidative stress increased by the Antarctic ozone hole (Mozzicafreddo et al., 2021).

Immunoblot analysis indicated the accumulation of DnaK protein (homolog of eukaryotic Hsp 70 that plays vital role in several abiotic stresses, including thermal stress) in the Antarctic psychrotroph *Shewanella* sp. Ac10 at 24°C (Yoshimune et al., 2005). The study also suggested that recombinant SheDnaK gene facilitates the growth of mutant *E. coli* at 15°C. Similarly, García-Descalzo et al. (2014) studied the thermal adaptation in Antarctic bacteria *Shewanella frigidimarina* towards different temperature ranges, from 0°C to 30°C. The significant accumulation in heat shock and other stress proteins is observed in bacterial cells cultured at 28°C (García-Descalzo et al., 2014).

Moreover, studies have indicated that psychrophiles at chilling temperature alter their metabolic pathways to conserve energy and thrive in cold environment. For long term survival, psychrophiles either down regulate their primary metabolic pathways (glycolysis, tricarboxylic acid (TCA) cycle, electron transport chain, pentose phosphate pathway etc.) or substitute them with abridged alternative pathways (glyoxylate, methylglyoxal, acetate metabolism, ethanol oxidation pathway etc.) (Collins and Margesin, 2019).

## Molecular adaptation

Molecular chaperones (RNA/DNA/protein) play an important role in stabilization of RNA/DNA and protein molecules at freezing temperature and in reducing protein aggregation and misfolding. These molecules are constitutively produced and expressed in psychrophiles as cold -adapted proteins. Several molecular chaperone proteins are identified from the Antarctic green alga *Chlamydomonas* sp. ICE-L shows high similarity with the genes of the psychrophilic bacterium *Psychroflexus torquis* and appears to be involved in tolerance to freezing, changing light and saline conditions in bacteria (Liu et al., 2016). Recently, Ijaq et al. (2022) investigated the functional role of hypothetical proteins from *Pseudomonas* sp. Lz4W, a Gram-negative psychrophilic bacterium adapted to survive in Antarctica. The study categorized two hypothetical proteins, HP AUB76544.1 and HP AUB76897, as chaperones that are important for correct folding and insertion of outer membrane proteins (HP AUB76544.1) as well as in sustaining the structural integrity and protein functions (HP AUB76897) (Ijaq et al., 2022). Furthermore, Solar Venero et al. (2022) studied the role of small non-coding RNAs (sRNAs) as genetic regulators in the adaptation of the Antarctic bacterium *Pseudomonas extremaustralis* towards changing climatic conditions and stress environment. The study showed the expression of novel sRNA, sRNA40 (identified by RNA-seq experiments) in response to different oxygen availability and oxidative stress and demonstrated that sRNA40 gene expression (associated to upregulation of selected secretory proteins) is triggered under aerobiosis/microaerobiosis conditions (Solar Venero et al., 2022).

Cold adapted enzymes are more flexible as compared to mesophilic enzymes due to the presence of relative low arginine content. Recently, one peroxidase named DyP (dye-decolorizing peroxidase) from an Antarctic bacteria *Pseudomonas* sp. AU10 was found to contain low arginine content (Cagide et al., 2023). Another study by Tang et al. (2019) characterized a cold-active alkaline pectate lyase from Antarctic bacterium *Massilia eurypsychrophila* with a low arginine content, suggesting that this is a distinctive property of cold adaptation. Petratos et al. (2020) studied the molecular dynamics of alcohol dehydrogenase (MoADH) from the cold-adapted bacterium *Moraxella* sp. TAE123 compared with the *Escherichia coli* (EcADH), *Geobacillus stearothermophilus* (GsADH), *Thermus* sp. ATN1 (ThADH). The cold adapted GH42 has also been identified in some microbes such as *Halobacterium lacusprofundi*, *Arthrobacter* sp. and *Marinomonas* sp. BSi20414 (Sheridan and Brenchley, 2000; Karan et al., 2013; Ding et al., 2017). Experimental study by Mangiagalli et al. (2021) on the cold adaptation of β-galactosidase from the psychrophilic *Marinomonas* ef1 shows the cold activity of this enzyme at 5°C and maintains stability up to 50°C, which suggests the origin of GH42 from the different evolutionary pathway. The biochemical and molecular features of GH42 and its biotechnological applications have been described in the recent review by Mangiagalli and Lotti (2021). Another study by Fan et al. (2016) reveals the structure of cold-active β-galactosidase from psychrotrophic *Rahnella* sp. R3. However, the glycoside hydrolase family (GH42) of other psychrophilic microbes is still poorly known. Thus, very little knowledge is available on the role of these enzymes in psychrophilic bacteria and adaptation to climate change in the Antarctic environment.

Another adaptation strategy by psychrophile bacteria is through horizontal gene transfer (HGT), which is considered as one of the important forces that regulates bacterial evolution (Martínez-Rosales et al., 2012). The occurrence of HGT was found in various Antarctic bacteria such as *Marinomonas* sp. ef1, *Pseudomonas* spp.*, Collimonas* sp. (Martínez-Rosales et al., 2012; John et al., 2020; Hwang et al., 2021). Recently, Abe et al. (2020) used neural network called Batch Learning Self-Organizing Maps BLSOM method to detect HGTs in the genomes of two Antarctic bacteria (*Sphingomonas* sp. HMP6 and HMP9) compared with other continents.

Though, the various adaptation strategies have been studied in Antarctic psychrophiles in response to changing climate as evident from the previous studies stated above. However, the research is still on the elementary phase and there are many unexplored areas including the underlying mechanism of adaptation, molecular pathways and key components involved which will give better understanding of Antarctic psychrophiles adaptation towards ongoing changing environment. Upcoming research on cold -adapted bacteria needs to be emphasized on these aspects by using novel techniques such as metagenomics and machine learning. These methods could serve as a dynamic tool to decipher adaptation mechanisms in the Antarctic psychrophiles, as they are difficult to culture in laboratory conditions.

## Industrial applications of Antarctic bacteria

### Cold active enzymes

The last few decades witnessed extensive research on microbial diversity inhabiting harsh extreme environmental conditions. Bacteria inhabiting extreme environments, like in Antarctica regions, are undergoing several stress factors and to withstand such conditions they have developed numerous physiological and molecular strategies (Hamid et al., 2022). These psychrophilic bacteria can survive and grow at a temperature range from −2 to 20°C, and express cold-adapted enzymes with distinctive properties that allow higher catalytic efficiency, improved flexibility, and lower thermal stability (Bruno et al., 2019). The advantages of cold-adapted enzymes over mesophilic enzymes are illustrated in various research articles (Kuddus, 2015; Javed and Qazi, 2016). Cold-active enzymes isolated from microorganisms are classified into three groups.

Group 1: Heat-sensitive with other enzymatic characteristics similar to mesophilic enzymes.
Group 2: Heat-sensitive and relatively more active than mesophilic enzymes at a low temperature.
Group 3: Same thermostability as mesophilic enzymes but more active than mesophilic enzymes at a low temperature.

**Figure 2:**
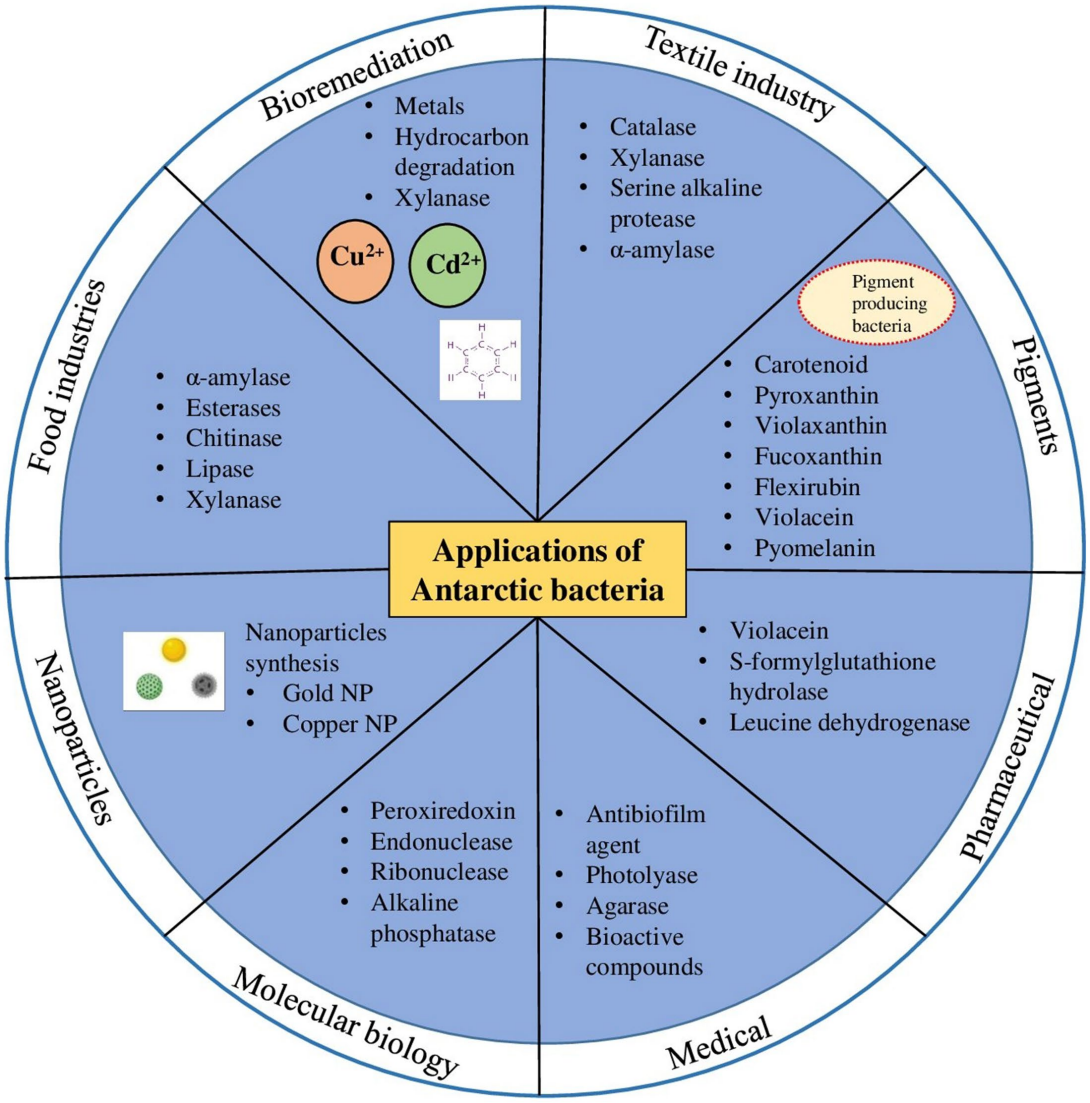
Biotechnological applications of cold-adapted molecules from Antarctic bacteria in different industrial sectors.

Since low temperatures are required for some industrial processes, there is a huge demand for cold-adapted enzymes in different biotechnological and industrial area, such as bioremediation, detergents, food and beverage processing, molecular biology and in textile industries (Figure 2) (Kuddus, 2018). Cold-active enzymes such as amylases, cellulases, lipases, pectinases, proteases, etc. from Antarctic bacteria establish an evident resource for several biotechnological applications. Many of the above enzymes are enormously used at commercial level, with special reference to hydrolases sourced from psychrophilic microorganisms (Hamid et al., 2022).

#### Food industry

Most of the Antarctic microbial enzymes have high catalytic competence at low and moderate temperatures when compared to mesophilic enzymes. This makes the cold-active enzymes unique in the food industry for their less biochemical requirements, reduction in process times, save energy costs and easy inactivation by gentle heat (Kuddus, 2018).

In dairy industries, the use of cold-active β-galactosidases is effectively operational in milk at lower temperatures that lead to lactose breakdown. Antarctic marine bacteria especially *Pseudoalteromonas haloplanktis*, has been proven to produce cold-active β-galactosidase with high efficacy in the hydrolysis of lactose under cold conditions (Cieśliński et al., 2005). Similarly, cold-active polygalacturonase isolated from *P. haloplanktis* can be used in juice manufacturing industries for degradation of pectin. Many other cold-active enzymes such as α-amylase, esterases, chitinases, and lipase have important applications in the food (Figure 2) and brewing industries, for cheese ripening, cheese flavoring, meat tenderizing, production of fatty acids and interesterification of fats, wheat bread making, improvement in food texture, etc. The gene encoding the cold active enzymes can be cloned into a suitable host to produce recombinant enzymes which are already proved to show high catalytic activity and stability at low temperatures (Table 2 and references therein). In case of food and brewage industries, low-temperature processing is highly favorable since it provides many advantages, for instances, prevention of bacterial contamination and occurrence of unwanted chemical responses, higher food quality, and persistence of flavor (Al-Maqtari et al., 2019).

**Table 2:** Polar-active enzymes isolated from Antarctic bacteria and their potential industrial applications (Reproduced from Bruno et al. (2019) and updated until recent literature).

Marine polar active enzymes	Organism source	Source of isolation	Potential industrial applications	References
EC3: Hydrolases				
β-galactosidase	*Pseudoalteromonas* sp. 22b	*Alimentary tract of Antarctic krill* Thyssanoessa macrura	Candidates for lactose removal from dairy products at low temperatures	Turkiewicz et al. (2003), Cieśliński et al. (2005)
	*Pseudoalteromonas haloplanktis* TAE 79	Antarctic seawater		Hoyoux et al. (2001)
	*Pseudoalteromonas haloplanktis* LMG P-1	Antarctic seawater		Gerday et al. (2000)
α-Amylase	*Pseudoalteromonas* sp. M175	Antarctic sea-ice	Detergent additive for stain removal in efficient way	Wang et al. (2018a)
	*Alteromonas* sp. TAC 240B	Antarctic seawater	Additives in processed food, in detergents for cold washing, in waste-water treatment, in bioremediation in cold climates and in molecular biology applications	Chessa et al. (1999)
	*Pseudoalteromonas haloplanktis*	Antarctic seawater		Feller et al. (1999), Kuddus et al. (2011)
Xylanase	*Flavobacterium frigidarium* sp.	Antarctic shallow-water marine sediment	Additives in textile and food industries, and bioremediation	Humphry et al. (2001)
*Serine protease (Subtilisin)*	*Bacillus* TA39	Antarctic seawater	Additives in low-temperature food processing, food and textile industries, leather processing, detergent industry	Narinx et al. (1992), Miyazaki et al. (2000)
	*Bacillus* TA41	Antarctic seawater		Davail et al. (1994), Miyazaki et al. (2000)
Serine protease	*Colwellia* sp. NJ341	Antarctic sea-ice		Wang et al. (2005)
Serine alkaline protease	*Shewanella* sp. Ac10u	Antarctic seawater		Kulakova et al. (1999)
Subtilisin-like serine protease	*Pseudoalteromonas* sp., *Marinobacter* sp., *Psychrobacter* sp., *Polaribacter* sp.	Antarctic seawater and thorax, abdomen and head of krill (*Euphausia superba* Dana)		Acevedo et al. (2013)
Protease	*Pseudoalteromonas* sp. NJ276	Antarctic sea-ice		Wang Q. et al. (2008)
	*Lysobacter sp. A03*	*Elephant Island, Antarctic*		Millan et al. (2022)
Aminopeptidase	*Pseudoalteromonas haloplanktis* TAC125	Antarctic seawater		De Pascale et al. (2010)
Serine peptidase	*Lysobacter* sp. A03	Penguin feathers in Antarctica		Pereira et al. (2017)
Metalloprotease	*Sphingomonas paucimobilis*	Stomach of Antarctic krill, *Euphausia superba* Dana		Turkiewicz et al. (1999)
	*Psychrobacter proteolyticus* sp. strain 116	Stomach of Antarctic krill, *Euphausia superba* Dana		Denner et al. (2001)
Lipase	*Pseudoalteromonas haloplanktis* TAC125	Antarctic seawater	Detergent additives used at low temperatures and biocatalysts for the biotransformation of heat-labile compounds	De Pascale et al. (2008)
	*Polaromonas vacuolata*	Antarctic seawater		Irgens et al. (1996)
	*Psychrobacter* sp.	Antarctic seawater		Parra et al. (2008), Xuezheng et al. (2010)
	*Shewanella frigidimarina*	Antarctic seawater		Parra et al. (2015)
	*Psychrobacter* sp. TA144	Antarctic seawater		Feller et al. (1991)
	*Psychrobacter* sp. 7,195	Antarctic deep-sea sediment (Prydz Bay)		Zhang et al. (2007)
	*Moritella* sp. 2–5–10-1	Antarctic deep-sea water		Yang et al. (2008)
	*Pseudoalteromonas* sp., *Psychrobacter* sp., *Vibrio* sp.	Antarctic seawater samples (Ross Sea)		Lo Giudice et al. (2006)
	*Moraxella sp.*	King George Island		Morozova et al. (2022)
	*Bacillus altitudinis Ant19 strain*	Antarctic soil	Detergent additives and useful for oil stain removal	Nimkande et al. (2023)
Esterase	*Oleispira antarctica*	Antarctic coastal water	Additives in laundry detergents and biocatalysts for the biotransformation of labile compounds at low temperatures	Lemak et al. (2012), Kube et al. (2013)
	*Pseudoalteromas haloplanktis* TAC125	Antarctic seawater		Aurilia et al. (2007), D’Auria et al. (2009)
	*Pseudoalteromas* sp. 643A	Alimentary tract of Antarctic krill *Euphasia superba*		Cieśliński et al. (2007)
Polyesterase	Moraxella sp.	Antarctic seawater	Bioremediation	Nikolaivits et al. (2022)
S-formylglutathione hydrolase	*Pseudoalteromonas haloplanktis* TAC125	Antarctic seawater	Candidates for chemical synthesis and industrial pharmaceutics	Alterio et al. (2010)
	*Shewanella frigidimarina*	Antarctic marine environment		Lee J. et al. (2019)
Polygalacturonas (pectin depolymerase)	*Pseudoalteromonas haloplanktis*	Antarctic seawater	Additive in food industries, such as clarification of juice, in the process of vinification, yield and color enhancement and in the mashing of fruits	Ramya and Pulicherla (2015)
RNA polymerase	*Pseudomonas syringae Lz4W*	Antarctic soil sample	Candidate for molecular biology application	Uma et al. (1999)
Cellulase	*Pseudoalteromonas haloplanktis*	Antarctic seawater	Additive in detergent industry	Violot et al. (2005)
	*Flavobacterium sp. AUG42*	Antarctic oligochaete	Food processing, textile processing and detergent industries	Herrera et al. (2019)
	*Nocardiopsis* sp.	Antarctic seawater	Cellulose hydrolysis efficiency	Sivasankar et al. (2022)
Alkaline phosphatase	TAB5 strain	Antarctica	Candidate for molecular biology application such as DNA dephosphorylation	Rina et al. (2000), Koutsioulis et al. (2008)
	*Shewanella* sp.	Intestine of Antarctic shellfish	Candidate for molecular biology application	Tsuruta et al. (2010)
	*Vibrio sp.*	North-Atlantic coastal waters	Candidate for molecular biology application	Hauksson et al. (2000)
	*Shewanella frigidimarina W32-2*	Antarctic sediments	Candidate for molecular biology application	Chen et al. (2021)
Acid phosphatase	*Shewanella frigidimarina W32-2*	Antarctic sediments	Candidate for molecular biology application	Chen et al. (2021)
Gelatin hydrolase	*Shewanella frigidimarina W32-2*	Antarctic sediments	Candidate for food and pharmaceutical industries	Chen et al. (2021)
Endonuclease (Cryonase)	*Shewanella sp. Ac10*	Antarctic seawater	Candidate for molecular biology application such as digestion of all types of DNA and RNA at cold temperatures (Takara-Clontech)	Takara-Clontech (n.d.)
Ribonuclease	*Psychrobacter* sp. ANT206	Antarctic sea-ice	Candidate for molecular biology applications	Wang et al. (2019)
Chitinase	*Arthrobacter psychrochitiniphilus, Arthrobacter cryoconite and Curtobacterium luteum*	Maritime Antarctica	Candidate for food industry, cosmetics and medicine	Vasquez et al. (2021)
EC1: Oxidoreductases				
Alcohol dehydrogenase	*Moraxella* sp. TAE123	Antarctic seawater	Candidate for asymmetric synthesis	Tsigos et al. (1998)
Aldehyde dehydrogenase	*Flavobacterium PL002*	Antarctic seawater	Potent catalyst for acetaldehyde determination in wine	Paun et al. (2022)
Alanine dehydrogenase	*Shewanella* sp. Ac10u, *Carnobacterium* sp. St2	Antarctic seawater	Candidate for enantioselective production of optically active amino acids	Galkin et al. (1999)
Leucine dehydrogenase	*Pseudoalteromonas* sp. ANT178	Antarctic sea-ice	Candidate for medical and pharmaceutical industry applications	Wang et al. (2018b)
Malate dehydrogenase	*Flavobacterium frigidimaris* KUC-1	Antarctic seawater	Candidate for detection and production of malate under cold conditions	Oikawa et al. (2005)
Superoxide dismutase	*Pseudoalteromonas haloplanktis*	Antarctic seawater	Candidates for applications in agriculture, cosmetics, food, healthcare products and medicines	Merlino et al. (2010)
	*Marinomonas* sp. NJ522	Antarctic sea-ice		Zheng et al. (2006)
	*Pseudoalteromonas* sp. ANT506	Antarctic sea-ice		Wang et al. (2017)
	*Halomonas sp. ANT108*	Antarctic sea-ice		Wang et al. (2020)
*Catalase*	*Bacillus* sp. N2a	Antarctic seawater	*Candidate for textile and cosmetic industry*	Wang W. et al. (2008), Sarmiento et al. (2015)
Glutathione reductase	*Colwellia psychrerythraea*	Antarctic seawater	Candidate as an antioxidant enzyme in heterologous systems	Ji et al. (2015)
Peroxiredoxin	*Psychrobacter* sp. ANT206	Antarctic sea-ice	Candidate for food and pharmaceutical industries	Wang et al. (2019)
Lignin peroxidase	Geobacter spp.	Antarctic soils	Bioenergy and bioremediation	Merino et al. (2021)
Manganese peroxidase	Geobacter spp.	Antarctic soils	Bioenergy and bioremediation	Merino et al. (2021)
Laccase	*Psychrobacter sp. NJ228*	Antarctic sea-ice	Candidate for Bioremediation	Zhang et al. (2022)
	*Halomonas sp. M68*	Antarctic seawater		Bisaccia et al. (2023)
Nitrate reductase	*Shewanella frigidimarina W32-2*	Antarctic sediments	Candidate for applications in agriculture and bioremediation	Chen et al. (2021)
Nitroreductase	*Psychrobacter sp. ANT206*	Antarctic sea-ice	Candidate for bioremediation	Wang J. et al. (2019)
EC6: Ligases				
DNA ligase	*Pseudoalteromonas haloplanktis* TAE 72	Antarctic seawater	Candidate for applications in molecular biology	Georlette et al. (2000)
EC4: Lyases				
γ-carbonic anhydrase	*Colwellia psychrerythraea*	Antarctic cold ice sediments	Candidates for biomedical applications	De Luca et al. (2016)
	*Pseudoalteromonas haloplanktis*	Antarctic seawater		De Luca et al. (2015), Angeli et al. (2019)
Pectate lyase	*Pseudoalteromonas haloplanktis* ANT/505	Antarctic sea-ice	Candidate for detergent industry	Sarmiento et al. (2015), Van Truong et al. (2001)
EC5: Isomerases				
Triose phosphate isomerase	*Pseudomonas* sp. π9	Antarctic sea-ice	Candidate for biocatalysis under low water conditions	See Too and Few (2010)
	*Moraxella* sp. TA137	Intestine of Antarctic fish		Rentier-Delrue et al. (1993)
CPD-photolyases	*Hymenobacter sp. UV11*	King George Island, Antarctica	Candidates for biomedical applications	Acosta et al. (2022)

#### Detergent industry

The removal of stains (lipids, polysaccharides, and proteins) by manual heating and beating of the clothes reduces the life of the fabrics and also decolorization (Kumar et al., 2021). On the other hand, harmful chemicals are most widely used in the chemical industries for removal of dirt which ultimately contaminates the environment. To overcome these issues, cold-active enzymes are the best alternative to the chemicals and moreover they increase the life of the fabrics, as manual heating is no longer required. The use of cold active enzymes such as lipases, amylases and proteases active at alkaline pH, with thermostability has solved the problems arising in the detergent industries. Switching mesophilic to cold-adaptive enzymes in the cleaning process ensures lowers wash temperatures and greater energy protection. A 10°C reduction in wash temperature created a 30% reduction in the consumption of electricity (Nielsen, 2005).

Proteases are found to be the most widely used enzymes in detergents and most of the cold-active proteases have shown remarkable stability and activity in a wide-ranging alkaline condition (Hamid et al., 2022). Cold-active proteases from *Bacillus* TA41, *Colwellia* sp. NJ341, *Pseudoalteromonas* sp. NJ276, *P. haloplanktis* etc. can be used as detergent additives for cold washing (Table 2 and references therein). The detergent manufacturers are seeking novel cold-active enzymes that may improve efficiency of detergents and retain the quality of fabrics (Al-Ghanayem and Joseph, 2020). Hence more studies on this aspect may lead to the discovery of many novel cold-active enzymes.

#### Molecular biology

Alkaline phosphatases which catalyze the hydrolysis of phosphate monoesters have a significant role in molecular cloning for dephosphorylation of DNA at the 5′ end to avoid its re-circularization. Based on this importance, New England Biolabs developed a recombinant alkaline phosphatase isolated from the Antarctic bacterial strain TAB5.^1^ In addition, cold active alkaline phosphatase from an Antarctic *Vibrio* sp., was reported to have a higher turnover number (k_cat_) and higher apparent Michaelis–Menten factor (K_m_) as compared with enzyme from *E. coli* (Hauksson et al., 2000). Similarly, to cold adapted alkaline phosphatases, uracil DNA N-glycosylases have been recently commercialized as a molecular biological tool by various companies (New England Biolabs Inc., Takara-Clontech, Affymetrix, Inc.) (Awazu et al., 2011; Muller-Greven et al., 2013).

DNA ligases are enzymes involved in DNA replication, DNA recombination and DNA repair. These enzymes are commonly used in molecular biology to catalyze the formation of a phosphodiester bond between adjacent 5′-phosphoryl and 3′-hydroxyl groups in double stranded DNA (Bruno et al., 2019). The well-established psychrophile *P. haloplanktis* TAE72, was also reported to produce DNA ligase and exhibit activity at temperatures as low as 4°C (Georlette et al., 2000).

#### Bioremediation

Bioremediation by mesophilic and thermophilic enzymes is ineffective in cold environmental conditions hence cold active enzymes are valuable tools for removal or biodegradation of pollutants. The use of cold-active enzymes could be more feasible and result oriented than the use of whole bacterial cells, since the whole cells requires multiple parameters of optimal growth (Kumar and Bharadvaja, 2019). Many cold adapted microorganisms, such as *Pseudomonas* sp., *Rhodococcus* sp., *Oleispira antarctica* and *Sphingomonas* spp. are proficient in degradation of petroleum hydrocarbons (Aislabie et al., 2006; Miri et al., 2019). Many cold active enzymes such as lipases, proteases, xylanase (Figure 2) etc. are regularly explored for various applications in bioremediation (Miri et al., 2019).

### Bacterial pigments

Pigments of natural origin play an important role in the physiology and molecular processes of microorganisms because they act as a strategy of adaptation to various extreme environments, have a protective function against solar radiation, and are also involved in functional processes like photosynthesis (Sutthiwong et al., 2014). Bacteria also produce a wide range of pigments such as carotenoids, melanin, violacein, prodigiosin, pyocyanin, actinorhodin, and zeaxanthin (Venil et al., 2013). Antarctica environment is also well known for richness of bacterial species producing various pigments such as carotenoids, flexirubin, violaceins, tetrapyrroles, quinones, biochromes, etc. (Figure 2).

Antarctic bacteria are able to produce not only different kinds of carotenoids but other pigments as well. This is supposedly due to the harsh conditions to which they are exposed. Therefore, these microorganisms may be regarded as promising potential targets for further research on the growing market of biotechnological pigments for industrial applications, not only focusing on long established compounds, but also on unconventional pigments. *Arthrobacter*, *Citricoccus*, and *Microbacterium* from the phylum Actinobacteria, *Chryseobacterium* and *Flavobacterium* from the phylum Bacteroidetes, and *Janthinobacterium*, *Pseudomonas*, *Lysobacter*, and *Serratia* from the phylum Proteobacteria are among the main pigment-producing bacteria reported in Antarctic environments (Silva et al., 2021 and references therein).

The carotenoid content, pyroxanthin, violaxanthin, fucoxanthin, and nostoxanthine 3-sulfate from the Antarctic bacterium *Pedobacter* showed strong antioxidant capacity and protects the bacterium against oxidative damage caused by high levels of UV-B radiation (Correa-Llanten et al., 2012).

Microbial pigments are of great interest in cosmetic, dairy, food, pharmaceutical and textile industries (Figure 2), mainly due to its new chemical structures and most importantly they are natural. A recent report from the global food colorants market showed that natural products represent one third of the total colorants approximately, and three fourths of these natural colorants are used in food in beverages.

## Other applications

Antarctic bacteria are a source of a wide range of applications in the medical sector due to their capacity of producing numerous compounds/metabolites (presence of diverse metabolic pathways resulting from evolutionary adaptation to subzero and nutrient deficient conditions) (Figure 2). Recently, a new anti-biofilm agent called “CATASAN” has been found in Antarctic bacteria *Psychrobacter* sp. TAE2020 which can be used against the human pathogen *Staphylococcus epidermidis* (D’Angelo et al., 2022). Similarly, another Antarctic bacteria *Pseudomonas* sp. TAE6080 is capable of inhibiting biofilm formation by the opportunistic pathogen *Staphylococcus epidermidis* (Riccardi et al., 2022). Several studies have shown that Antarctic microbes produce bioactive compounds to treat various diseases (Murray et al., 2021; Xiao et al., 2023). Quinones from orange-yellow pigmented *Sphingomonas aerolata* (Busse et al., 2003), isolated from the ice of Taylor dome and hydrocarbon-contaminated soils around Scott Base is used to treat Alzheimer’s, Huntington’s, Parkinson’s, and cardiovascular diseases (Nair and Abraham, 2020). Previous analysis showcased that the Antarctic pigments also have various biological activities such as antioxidant, antibacterial, antimalarial, antifungal, anticancer and many others (Silva et al., 2021). Experimental studies by Maeda et al. (2009) on fucoxanthin (characteristic carotenoid of brown algae) have revealed various applications of the compound in producing anti lymphangiogenic, antitumoral, neuroprotective, antidiabetic, anti-obesity, and anti-inflammatory effects. In addition, fucoxanthin prevents carcinogenesis and depressive behavior, such as the attenuation of bleomycin-induced lung fibrosis and ulcerative colitis (Wang Y. et al., 2019). Another pigment violaxanthin has proved to have antiproliferative and anti-inflammatory effects (Pasquet et al., 2011; Soontornchaiboon et al., 2012). Decaprenoxanthin from *Arthrobacter psychrochitiniphilus* strain 366 isolated from a biofilm formed on the surface of defrost water in Whalers Bay, Deception Island, Silva et al. (2019) has strong antioxidant properties. Apart from pigments, a variety of antimicrobial lipid-based substances have been isolated from Antarctic microorganisms with potential to be used in treatments of bacterial infections. Rhamnolipids, a special class of bacterial lipids purified form Antarctic marine sediment bacteria, *Pseudomonas* sp. BTN1 displayed antibacterial activity against *Burkholderia cenocepacia* (isolated from cystic fibrosis patient) and *Staphylococcus aureus* (Tedesco et al., 2016). Aminolipids, another category of microbial lipids purified from shallow-sea-sediment bacterium *Aequorivita* sp. is effective against methycilin-resistant *Staphylococcus aureus* (Chianese et al., 2018).

Besides bioactive compounds, Antarctic bacteria synthesizes metal nanoparticles (Figure 2) through biomineralization process. The biosynthesis of metal nanoparticles using Antarctic bacteria is a cost-effective, environmentally friendly process, without using toxic chemicals in the synthesis and purification steps. In recent years, synthesis of metal nanoparticles using cold adapted bacterial strains have gained attention due to the high stability (even at psychrophilic conditions) and diverse biomedical applications (Das et al., 2020; John et al., 2021, 2022). Das et al. (2020) biosynthesize gold nanoparticle (GNP) at different temperatures (4°, 10°, 25°, 30° and 37° C) using psychrotolerant Antarctic bacteria *Bacillus* sp. GL1.3. The synthesized gold nanoparticles exhibit antibacterial activity against sulfate-reducing bacteria (*Desulfovibrio* sp.) (Das et al., 2020). A similar study by Javani et al. (2015) identified four psychrophilic Antarctic bacteria namely *Aeromonas salmonicida*, *Pseudomonas veronii*, *Psychrobacter* sp. and *Yersinia kristensenii* that extracellularly biosynthesize nanosilver at 4°C and 30°C. The study demonstrated that the most active and stable nanoparticles with highest antibacterial activity were those prepared at 4°C. These nanoparticles possess high stability even after 10 months of incubation under light (Javani et al., 2015). An efficient novel approach is used by Plaza et al. (2016) to synthesize quantum dots (CdS and CdTe quantum dots) at room temperature by using heavy metal (cadmium and tellurite) resistant Antarctic bacteria, *Pseudomonas*, *Psychrobacter* and *Shewanella*. Recently, John et al. (2022) synthesizes silver nanoparticles (AgNPs) using three bacterial strains *Rhodococcus*, *Brevundimonas* and *Bacillus* isolated from an Antarctic consortium. Biosynthetic AgNPs show promising effects against common nosocomial pathogens and can be replaced with conventional antibiotics (John et al., 2022). Thus, despite being underexplored, Antarctic bacteria constitute a promising platform for biosynthesis of nanomaterials.

## Advanced strategies to study Antarctic bacterial adaptation

### Omics

**Figure 3:**
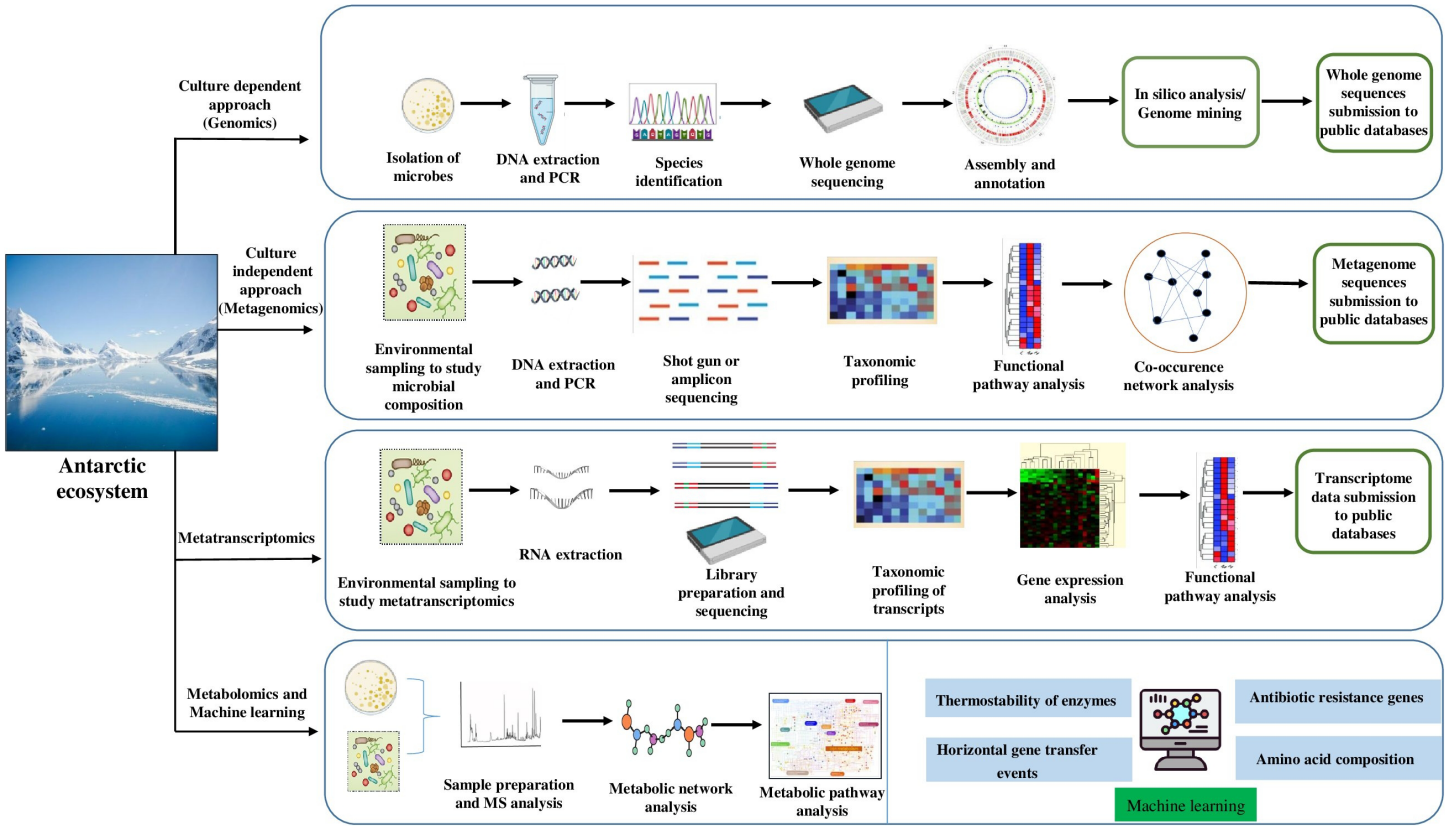
Advanced technologies – omics tools and machine learning approach to study the adaptation mechanism and potential of cold-adaptive molecules of Antarctic bacteria.

To gain a comprehensive picture of bacterial communities, several “omics” approaches should be applied to reveal polar “blackbox” microbes (Figure 3). The development of genomic technologies in recent years has gained knowledge on microbial communities and their adaptation in the Antarctic ecosystem. The most common technique to reveal the taxonomical composition of cold adapted bacteria is 16S rRNA gene sequencing (Bowman and Ducklow, 2015), but the functional role of many other genes remains unknown. However, new technologies are being developed in high -throughput sequencing, which provides high quality data with short or long read sequences. To date, several genomes from psychrophilic bacteria and archaea have been sequenced (Pucciarelli et al., 2015; Ramasamy et al., 2019; John et al., 2020; Wang et al., 2021; Lee et al., 2022; Riccardi et al., 2022; Otur et al., 2023). The advantage of whole genome sequencing of Antarctic bacteria is to analyze and characterize the genes in the entire genome, especially genes coding industrially relevant enzymes using DNA sequencing methods and bioinformatics tools (assemble and analyze the structure and functions of specific gene). However, the whole genome is limited to cultivable bacteria which can grow as pure cultures in the laboratory conditions (Figure 3). Due to lack of cultivation methods in laboratory conditions, the majority of bacteria on our planet are uncultured and hence unidentified (Hug et al., 2016).

Up till now the whole genome sequence of Antarctic bacteria is available for a few taxa. The advancement in sequencing methods and bioinformatic approach could provide the understanding of their physiological and metabolic roles. The portable sequencers of Oxford Nanopore Technologies MinION could be used as an *in situ* sequencing tool for community composition and functional profiling of microbes thrive in Antarctic environment. Although, the high error rate of Nanopore sequencing compared to the amplicon sequencing technology by the Illumina platform is gradually decreasing, the hybrid assembly strategy (i.e., Illumina short reads assembled together with Nanopore long reads) is considered the best to cover the novel taxa and their functions in various Antarctic microbial communities.

The use of long-read metagenomic sequencing by Waschulin et al. (2022) revealed the biosynthetic potential of uncultured bacterial phyla such as *Acidobacteriota*, *Verrucomicrobiota* and *Gemmatimonadota*. Additionally, the uncultivable bacteria and their genetic functions can be explored through the metagenomic approach (genetic material of mixed community directly recovered from natural environment without obtaining the pure culture) which can either be sequence based, including high-throughput sequencing and bioinformatic analysis (high quality metagenome-assembled genomes, obtained through combination of binning approaches), or function based by involving functional expression of metagenomic libraries to identify target genes/gene clusters (Figure 3) (Alneberg et al., 2018).

Uncultivated microbial clades (candidate phyla) belonging to Genome Taxonomy Database (GTDB) in the Antarctic Ace Lake may play an important role in nutrient cycling (Williams et al., 2022). Similar studies have been reported by Williams et al. (2021) on microbial “dark matter” i.e. *Candidatus* bacterial phyla of metagenome-assembled genomes (MAGs) obtained from an Antarctic Lake. Recently, Fonseca et al. (2022) identified the bacterial family *Woeseiaceae* for the first time in Antarctic sediments, but the cellular and molecular adaptation of this family to the cold environment is unknown. Although the metagenomic approach reveals the taxonomic composition, to deeply understand the expression of genes in the microbes to environmental changes, the metatranscriptome provides the information about microbial functions associated with the environment (Sutherland et al., 2022). The functional diversity of the microbial communities has been recently investigated under the Antarctic ice shelf using multiomics approach such as metagenomics, metatranscriptomics, single-cell genomics by Martínez-Pérez et al. (2022).

A study by Médigue et al. (2005) sequenced the genome of the Antarctic bacterium *Pseudoalteromonas haloplanktis* TAC125 and using *in silico* analysis revealed the composition of the proteome for cold adaptation. Similarly, Fondi et al. (2015) investigated several metabolic features of *Pseudoalteromonas haloplanktis* TAC125 and variations in cellular metabolic fluxes through *in silico* modeling.

Recent developments in the omics era will open a remarkable milestone in structural and functional metagenomics (Prayogo et al., 2020). In fact, metagenome mining is applied to bacterial communities to screen novel classes of cold adapted enzymes for biotechnological applications (Kumar et al., 2021). A recent study by Blázquez-Sánchez et al. (2022) found Antarctic bacteria *Moraxella* sp. strain TA144 (Mors1) and *Oleispira antarctica* RB-8 (OaCut) hydrolyze aliphatic and aromatic polyesters at moderate temperatures. In addition, metagenome analysis revealed the members of the *Moraxellaceae* family harbors candidate genes for polyethylene terephthalate (PET) hydrolases (Blázquez-Sánchez et al., 2022). The majority of bacteria living in the cold expresses certain genes as adaptation strategies. Dall Agnol et al. (2014), using transcriptomes (Figure 3) and proteomes, unveiled global gene expression in response to thermal adaptation of *E. antarcticum* B7 at different temperatures (0°C and 37°C). In a recent study by Ijaq et al. (2022), *Pseudomonas* sp. Lz4W genome analysis revealed 743 CDS annotated as hypothetical proteins, including 61 hypothetical proteins at translational level. However, with the current global climate crisis, there is the urgent need to understand all the adaptation mechanisms to the changing environmental conditions, particularly in the polar region. Recent developments in proteomics along with gene expression profiling have been coupled for the discovery of various biomolecules in psychrophilic bacteria. Garcia-Lopez et al. (2021) presented a review of the current knowledge on psychrophiles for their biomolecules and metabolic pathways. Understanding the adaptation strategies of Antarctic bacteria using the omics approach will help to reveal their metabolic changes (Figure 3) to future climate change scenario.

### Machine learning approach

Machine learning (ML) is a branch of artificial intelligence (AI) that designs mathematical models to execute certain tasks from assembled information in less time and cost. Generally, two ML models – supervised (also known as predictive) and unsupervised (descriptive)- have been extensively used in most research areas in the field of microbiology (Goodswen et al., 2021; Greener et al., 2022). The supervised model requires to be trained using the training data set which includes text, images, and alphanumeric data. The most used supervised model algorithms are classification and regression. While the unsupervised algorithm uses unlabeled data which involves clustering and association rule mining (Goodswen et al., 2021). The ML approach has evolved rapidly in recent years to understand microbial and molecular processes using high-throughput data. Considering the various applications of this approach in biology, very scarce studies are available for psychrophilic microbes, especially bacteria. Previously, Lee C. et al. (2019) used ML method (classification and regression tree machine learning algorithms called CART) to study the bacterial communities and geochemical variables. The study provides a clue to unravel the bacterial communities link to changing environmental conditions using ML approach in other habitats of the Antarctic environment. Later, the mobilized colistin resistance (*mcr*) gene, that is a type of antibiotic resistance gene (ARG), has been identified using machine learning tools (Figure 3) in polar *Psychrobacter* (Cuadrat et al., 2020). Similarly, Arango-Argoty et al. (2018) developed a tool called DeepARG using a deep learning approach for the prediction of antibiotic resistance genes from metagenomic data. Recent study by Marcoleta et al. (2022) employ DeepARG tool to detect ARG from North Antarctic soils microbial communities (*Pseudomonas*, *Streptomyces*, *Gemmatimonas*, *Paenibacillus*, and *Polaromonas*). Yet, sparse information is available for antibiotic resistance genes (ARG) of resistome profile of Antarctic bacteria. Moreover, genomic, and metabolic pathways of novel taxa of bacteria are poorly known. The ML is a promising approach and can be widely used in omics data to explore the presence of ARGs in Antarctic habitats.

Furthermore, studies based on machine learning have been reported on thermophilic and mesophilic bacterial proteins, however little is known about the application of ML approach in psychrophilic enzymes. Recent study on psychrophilic amino acid composition (AAC) using machine learning (ML) algorithm showed that psychrophiles proteins contains high frequency of Ala, Gly, Ser, and Thr, compared to Glu, Lys, Arg, Ile, Val, and Leu amino acids (Huang et al., 2023a). The support vector machine learning model (SVM) in combination with molecular dynamics (MD) is employed to study the thermostability of psychrophilic alpha-amylase (Figure 3) (exhibited high activity at low temperature) isolated from *Pseudoalteromonas haloplanktis* (Li et al., 2020). The study revealed the presence of two single point mutations (S255K and S340P) and one double mutation (S255K/S340P) at non-conserved residues that enhanced thermostability of enzyme without altering its catalytic activity (Li et al., 2020). Similarly, to understand the antifreeze peptides and proteins interactions to ice crystals, several computational predictors have been used (Jiang et al., 2022). Recently, ML-guided robotic strain isolation platform for the isolation of diverse microbes from human feces have been used by Huang et al. (2023b). These ML approaches can be applicable to bridge the knowledge gap on genes functions in bacterial genome in response to climate change adaptation (Figure 3). Hence, we think this algorithm can be extendable to the Antarctic ecosystem in the upcoming studies to develop the understanding on bacterial dark matter and their adaptation.

ML has the potential to accelerate HIV drug discovery by narrowing down the number of antiviral compounds selected for *in vitro* and *in vivo* testing.

## Conclusion and future perspectives

The cold-adapted Antarctic psychrophilic bacteria represent excellent model organisms to study climate change induced stress adaptation. These bacteria are thriving in harsh and inhospitable Antarctic environment and displaying immense potential of regulating climate change factors. Therefore, the comprehensive review on Antarctic bacteria highlighted the adaptation strategies of psychrophiles at various levels (structural, physiological and molecular) in response to the changing environment. Many of these adaptation tools including biosurfactants, EPS, PUFA, membrane pigments, molecular chaperones and the underlying mechanisms are superficially studied due to the difficulty in some bacterial cultivation. The aid of ML and omics approach, particularly metagenomics, could provide insight into how psychrophilic bacteria adapt to cope with cellular and molecular mechanisms as survival strategies. Extensive research using these approaches needs to be done to better understand the bacterial adaptation in Antarctic environment and implementing this knowledge for improving the tolerance ability of other bacteria, for an environmentally sustainable future. The multi-omics tools coupled with ML algorithms might explore the industrial potential of biomolecules from Antarctic bacteria effectively in less time and labor. This is another aspect of upcoming research that needs to be emphasized. In recent years, the exploitation of psychrophilic enzymes (amylase, lipase, protease, hydrolase, pectinase, cellulase etc.) has been increased in different biotechnological industries due to their improved catalytic efficiency, flexibility, and low thermal stability. The cold-active enzymes are the best eco-friendly alternative to synthetic chemicals and moreover it increases the shelf life of the products (fabrics, foods etc.). Therefore, the manufacturers are seeking novel cold-active enzymes that further improve the efficiency and quality of products (particularly in the detergents industry). Hence, this is an additional aspect of research that needs to be explored. Overall, the review discusses the most recent studies on Antarctic bacterial adaptation as future climate model and suggested novel approaches for upcoming research in this direction.

## Author contributions

KPR and LM conceptualized the idea and constructed figures. RR and KPR constructed tables. KPR, LM, RR, and KR wrote the original draft. CM and SP revised the manuscript. KPR wrote the final draft with all other authors. All authors contributed to the article and approved the submitted version.

## Funding

The authors are thankful to Umeå University for the funding provided for Open access fee.

## Conflict of interest

The authors declare that the research was conducted in the absence of any commercial or financial relationships that could be construed as a potential conflict of interest.

## Publisher’s note

All claims expressed in this article are solely those of the authors and do not necessarily represent those of their affiliated organizations, or those of the publisher, the editors and the reviewers. Any product that may be evaluated in this article, or claim that may be made by its manufacturer, is not guaranteed or endorsed by the publisher.

## Footnotes

^1^ https://www.neb.com/products/m0289-antarctic-phosphatase#Product%20Information
